# β-Caryophyllene Ameliorates Cyclophosphamide Induced Cardiac Injury: The Association of TLR4/NFκB and Nrf2/HO1/NQO1 Pathways

**DOI:** 10.3390/jcdd9050133

**Published:** 2022-04-26

**Authors:** Nancy S. Younis

**Affiliations:** Department of Pharmaceutical Sciences, College of Clinical Pharmacy, King Faisal University, Al-Ahsa 31982, Saudi Arabia; nyounis@kfu.edu.sa; Tel.: +966-547045757

**Keywords:** β-caryophyllene, cyclophosphamide, cardiotoxicity, Toll like receptor 4

## Abstract

Background: β-caryophyllene (BCP), a natural sesquiterpene, is extensively present in the essential oils of several plants. Cyclophosphamide (CYC) is an anticancer drug. However, its clinical usage is inadequate due to its cardiotoxicity. The aim of this study was to study the effects of BCP on cardiac injury induced by CYC exposure, and to identify the underlying mechanism of action. Methods: Five groups of Wistar rats were allocated. Group I (Normal), II (BCP), and III (CYC) acted as controls. Group IV, V (CYC + BCP) received BCP in two doses (100 and 200 mg/kg, orally, respectively) for 14 days after CYC challenge. CYC groups received 200 mg/kg, i.p. of the drug once on the first day of experiments. Results: CYC group displayed numerous ECG and histological irregularities and cardiac markers elevation. CYC induced lipid peroxidation and oxidative stress intensification, as well as inflammatory and apoptotic markers escalation. Treatment with BCP resulted in modified ECG traces and histological sections. BCP mitigated cardiac markers and lipid peroxidation whereas intensified antioxidant capacity. BCP activated Nrf2, with subsequent HO1 and NQO1 amplification. BCP diminished TLR4/NFκB pathway, which consequently lessened the inflammatory and apoptosis responses. Conclusion: BCP administration was associated with activated Nrf2/HO1/NQO1 and inhibited TLR4/NFκB pathways with subsequent enhanced anti-oxidative capacity and diminished inflammatory and apoptosis responses.

## 1. Introduction

β-caryophyllene (BCP) is extensively dispersed in numerous essential oils isolated from different plants and contributes to the exclusive scent of some plant essential oils, e.g., cloves, cinnamon, and basil [1]. BCP is approved by the FDA as a food additive, and is considered as a “generally recognized as safe” food or cosmetic addition [2]. BCP, a bicyclic sesquiterpene, displayed numerous pharmacological actions, including antioxidant [3], anti-inflammatory [4], anticancer, analgesic [5], anticonvulsant [6], lipid lowering [7], and antispasmodic [8] effects. Furthermore, BCP demonstrated antioxidant, anti-inflammatory, and re-epithelialization activities in a rat skin wound excision model [9], an antidepressant-like effect in restraint plus stress-induced depression [10], improved the systemic inflammation and oxidative status of arthritic rats [11], and protected rat from carbon tetrachloride-induced hepatic fibrosis [3]. Moreover, β-Caryophyllene attenuated hyperoxaluria-induced kidney defective function [12], ameliorated cisplatin [13], and sulfasalazine-induced nephrotoxicity [10]. BCP exerts numerous cardiac actions. For instance, BCP reduced atherogenic index and coronary risk index in hypercholesterolemic rats [14], ameliorated isoproterenol (ISO)-induced myocardial infraction [15,16] via a cannabinoid receptor-2 (CB2) dependent and independent manner [17], and mitigated doxorubicin induced acute cardiotoxicity [18]. BCP has been identified as a fully selective agonist of CB2 cannabinoid receptors, which is one of the key members of the endocannabinoid system (ECS) [4]. Other than CB2 receptors, BCP was established to interact with peroxisome proliferator-activated receptor PPAR α and γ [1], TLR4/NFκB [15], MAPK [19], Nrf2/HO1 [20], and PI3K/Akt [21], among other signaling pathways.

Cyclophosphamide (CYC) is an alkylating anticancer that is used clinically in bone marrow transplantation, rheumatoid arthritis, lupus erythematosus, multiple sclerosis, and different types of cancer [22]. However, CYC induces dose-related cardiotoxicity, which limits its usage clinically, with mortality rate extended from 11% to 43% even at the therapeutic dose [23]. CYC is associated with cardiomyocyte apoptosis, inflammation, endothelial dysfunction, calcium dysregulation, endoplasmic reticulum damage, and mitochondrial damage [24]. Several molecular mechanisms were proposed discussing CYC induced cardiotoxicity, e.g., through Nrf2/HO-1 [25,26], PI3K/Akt/mTOR [25,26], SIRT1/FoxO1 [27], Akt/GSK-3β/NFAT/calcineurin [22], p53/p38MAPK [26], and TLR4/NFκB [19,28], among other signaling pathways. CYC undergoes hepatic metabolism to produce of aldophosphamide, which further decomposes into phosphoramide mustard, the active neoplastic metabolite, and acrolein, the detrimental metabolite [29]. Acrolein is responsible for cardiotoxicity via exhaustion of antioxidants/ATP level, alteration in contractility. Furthermore, acrolein causes injured endothelium and augmented pro-inflammatory/pro-apoptotic actions, and consequently, cardiac remolding and failure as well as myocardial infarction may happen [24]. Acrolein prompts reactive oxygen species (ROS) production, such as superoxide radicals and hydrogen peroxide [30], causing injury to the inner mitochondrial membrane, thus weakening the oxygen radical detoxifying ability [31], resulting in cardiac injury [24]. Thus, the enhanced elimination of acrolein as well as augmentation of endogenous antioxidants could be a beneficial approach to mitigate CYC toxicities.

Previous studies have shown that BCP is able to activate the cellular antioxidant system and inhibit inflammatory gene expression, consequently protecting against oxidant or inflammatory induced organs damage. However, it is unknown whether BCP influence CYC induced cardiac injury or not. We hypothesized that BCP may protect against cardiac injury. To investigate this hypothesis, we examined the effects of BCP on cardiac injury induced by CYC exposure and attempted to find a possible mechanism of action.

## 2. Materials and Methods

### 2.1. Ethical Approval Statement

Animal treatment and experiments implementation were in agreement with the procedures and regulations of the Ethical Conduct for Use of Animals in Research in King Faisal University. The experimental protocol was allowed by Institutional Animal Care and Use Committee of the King Faisal University with the ethical agreement no KFU-REC-2022-FEB-EA000417.

### 2.2. Animals’ Experimental Protocol

Male Wistar rats aged 6–8 weeks (weight 190–220 g) were procured from Experimental Animal Research Centre, King Saud University, Saudi Arabia. Rats were maintained in ventilated cages system (12 h light/dark cycle, 20.3–23.1 °C) and continued on standard laboratory food and water ad libitum during the entire study.

After one week acclimation, rats were divided randomly into five groups of 6 animals each. Group I (Normal) served as normal control and received saline for 14 days. Group II (BCP) received β-caryophyllene (BCP) (200 mg/kg body weight, orally) for 14 days [23]. Group III (CYC) served cyclophosphamide (CYC) control, in which rats received one injection of CYC (200 mg/kg, i.p.) on the first day of experiments to induce cardiac injury [32]. Group IV, V (CYC + BCP) received CYC (200 mg/kg, i.p.) on the first day and BCP (100 and 200 mg/kg, orally) for 14 days [15].

### 2.3. Electrocardiogram (ECG) Recording and Measurement

At the last day of the experiment, rats were anaesthetized using urethane (1.5 g kg^−1^). Rat were placed in prone position on the ECG platform of Emka IOX data acquisition software, with both fore and hind limbs taped to the leads. Uninterrupted ECG recordings were obtained and analyzed using ECG analyzer software from Emka Technologies’ systems (France). The changes in P-R interval in millisecond (ms), R-R interval (ms), Q-T interval (ms), R wave amplitude (mV), and heart rate HR (beat/min) were determined electronically.

### 2.4. Sample Collection

At the end of the experiment and after ECG measurement, blood samples were obtained from abdominal aorta of the anaesthetized rat. Sera were collected and stored −80 °C for subsequent biochemical analysis. Tissues samples were freshly frozen in liquid nitrogen and transferred to a −80 °C freezer until used.

### 2.5. Histological Analysis

Samples for histological analysis were fixed in 10% formaldehyde at room temperature and embedded in paraffin blocks using a Leica Microsystem tissue processor (ASP 300S, Munich, Germany). For histological staining, sections of 3 μm thickness were sliced using a Leica Microsystem microtome (Model RM 2265, Munich, Germany). Cardiac sections were stained using Masson’s trichrome and hematoxylin-eosin staining (H&E) and examined blindly under light microscope. Collagen area was estimated using Masson’s trichrome stained slides as mentioned before [32].

### 2.6. Cardiac Enzymes Measurement

Cardiac enzymes, including Creatine Kinase Myocardial Bound (CK-MB, cat no. 8.05.13.0.0100, Atlas Medical, Cambridge, UK), Creatine Phosphokinase and Lactate Dehydrogenase (CPK, LDH, cat no. ab155901, ab102526 respectively, Abcam Inc., Waltham, MA, USA), and Aspartate Aminotransferase (AST, cat no. MBS2540582, MyBioSource, San Diego, CA, USA) were measured as mentioned in the manufacturer’s procedure.

### 2.7. Antioxidant Activities and Lipid Peroxidation Measurement

Malondialdehyde (MDA, cat no. ab118970), Superoxide Dismutase (SOD, ab65354), Catalase (CAT, cat no. ab83464), Glutathione reductase (GRx, cat no. ab239727), Glutathione Peroxidase (GPx, cat no. ab102530), and Hydrogen Peroxide (H_2_O_2_, cat no. ab102500) assay kits were obtained from Abcam Inc. Waltham, MA, USA.

### 2.8. Inflammatory Mediators Measurement

Tumor Necrosis Factor-α (TNF-α, cat no. ab46070), Interleukin-1β (IL-1β, cat no. ab100768), Interleukin-6 (IL-6, cat no. ab100772), and Nuclear Factor κB (NFκB/p65, cat no. ab133112) ELISA kits were all purchased from Abcam Inc. Waltham, MA, USA.

### 2.9. Quantitative Real-Time Polymerase Chain Reaction (PCR) Technique

Real-time PCR was performed according to the technique described elsewhere [15] to evaluate Toll like receptor 4 (TLR 4), nuclear factor erythroid 2-related factor 2 (Nrf2), heme oxygenase 1 (HO1), NAD(P)H quinone dehydrogenase 1 (NQO1), Bax, and Bcl2. Quantification analyses were completed using an Opticon-2 Real-time PCR reactor (MJ Research, Reno, NV, USA). Step PE Applied Biosystems (PerkinElmer, Waltham, MA, USA) software was used to analyze real-time PCR results. Expression of the target gene was measured and quantitated by the reference gene (β-actin). The primer sequences utilized in this study were as follows: TLR 4 F: 5′ CATGACATCCCTTATTCAACCAAG 3′ and R: 5′ GCCATGCCTTGTCTTCAATTG 3′; Nrf2 F: 5′ CATTTGTAGATGACCATGATC GC 3′, R: 5′ ATCAGGGGTGGTGAAGACTG 3′; HO-1 F: 5′ GTGCACATCGTGCAGAA 3′, R: 5′ GTGCACATCCGTGCAGAGAA 3′; NQO1: F: 5′ AGGATGGGAGGTACTCGATC 3′ and R: 5′ AGGCGTCCTTCCTTATATGCTA 3′; Bax F: 5′ GTGGTTGCCCTCTTCTACTTT G 3′, R: 5′ CAAAAGATGGTCACTGTCTGC 3′; Bcl-2 F: 5′ CCGGGAGATCGTGATGAAG T 3′, R: 5′ ATCCCAGCCTCCGTTATCCT 3′ and β-Actin F: 5′ CACGATGGAGGGGCCGA CTCATC 3′, R: 5′ ATCCCAGCCTCCGTTCCAACAGT 3′.

### 2.10. Evaluation of Nrf2/HO1/NQO1 Pathway Protein Expression

Western blot was preformed according to the method described previously [15]. Briefly, cardiac samples were homogenized with radioimmunoprecipitation assay (RIPA) buffer inclosing protease inhibitor. The concentration of total protein extracted was calculated using a NanoDrop Lite spectrophotometer (Thermo Fisher Scientific, Waltham, MA, USA). Afterward, 50 µg of the total extracted protein was separated via sodium dodecyl sulfate (SDS)-polyacrylamide gel electrophoresis (PAGE) and blotted onto PVDF membranes. Blocking PVDF membranes were achieved by incubation in Tris-buffered saline (TBS) enclosing 3% bovine serum albumin and 0.1% Tween 20 for 1 h at room temperature. After washing with TBS containing 0.1% Tween 20, the membranes were incubated firstly with the primary antibodies (1:300 dilution) for 2 h, and then goat anti-rabbit HRP-conjugated (as secondary antibody; at a 1:5000 dilution) at room temperature. The primary antibodies used were as follows: Anti-Nrf2 (1:1000), anti-HO-1 (1:1000), anti-NQO-1 (1:1000), and β-actin (1:1000) obtained from Cell Signalling Technology, MA, USA. The chemiluminescence produced from the luminol reagent was detected with the C-DiGit chemiluminescence scanner (LI-COR, Lincoln, NE, USA), and the band intensity was analyzed using the scanner software.

### 2.11. Immunohistochemical (IHC) Analysis

Cardiac sections were used for IHC staining. Hence, 3% hydrogen peroxide (H_2_O_2_) in methanol was used to block the endogenous peroxidase enzyme in the obtained sections at 21–25 °C for 30 min, followed by rinsing three times in phosphate-buffered saline (PBS). Afterward, the sections were incubated with the antibodies, stored overnight at 4 °C in a humidified chamber, and then goat anti-rabbit-horseradish peroxidase (HRP)-conjugated IgG antibody (1:1000; cat. no. ab6721; Abcam) was added for 1 h at 37 °C. The primary antibodies against rabbit polyclonal anti NF-κBp65 antibody (Thermo Fisher Scientific, USA), and anti-TLR4 antibody (Abcam, UK), were used following the procedures described previously [33]. Finally, the sections were developed with 1% diaminobenzidine for 5 min, counterstained with 1% hematoxylin for 2 min at 21–25 °C, and mounted with neutral gum. Cardiac slices were imaged using a microscope fitted with a digital camera (Nikon Instruments Inc., Tokyo, Japan). NIS-Elements software was used for the semi-quantitative analysis of NFκB and TLR4. First, the area of the immunohistochemical reaction in the picture was selected. Then, the average optical density in the selected area of each picture was measured. Positive cells were counted under 400× magnification observing 10 consecutive non-overlapping fields per animal in a blinded manner

### 2.12. Statistical Analysis

The results are expressed as mean ± SD. Statistical significance was assessed using one-way analysis of variance (ANOVA), followed by the Tukey–Kramer multiple comparison tests. A level of *p* < 0.05 was considered significant

## 3. Results

### 3.1. β-Caryophyllene Attenuated CYC Induced Fluctuations in Body Weight, Heart Weight and Heart to Body Ratio

At the end of study, normal and BCP control animals exhibited regular respiration, and quick reflexes, whereas CYC animals displayed weakness, short, shallow breathing, and diarrhea. These observations were minimized in BCP treated groups. Furthermore, CYC group displayed diminished body weight and increased heart to body ratio significantly when related to normal animals. Meanwhile, treatment with BCP caused a significant increase in the body weight and decreased the heart weight and heart to body weights ratio when related to CYC animals (Table 1, *p* < 0.05).

### 3.2. β-Caryophyllene Attenuated CYC Induced Fluctuations in ECG and Heart Rate

**Figure 1:**
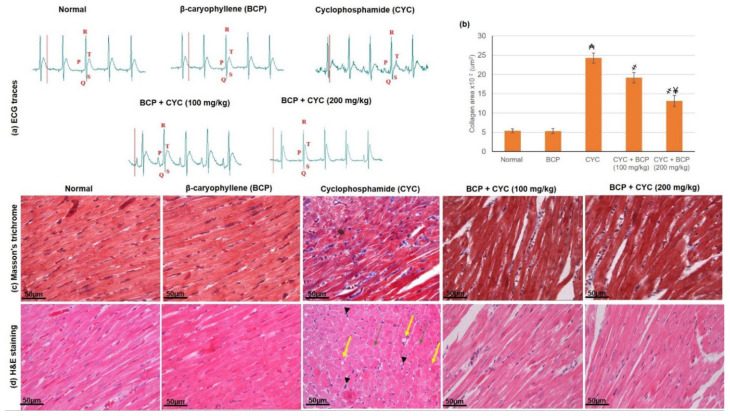
Effects of treatment with β-caryophyllene (BCP) for 14 days on cyclophosphamide (CYC) induced cardiac injury on (**a**) ECG traces, (**b**) Quantification of collagen area from Masson trichrome stained sections and on heart sections stained with (**c**) Masson’s trichrome, (**d**) H&E in rats. All values are expressed as mean ± SD. ₳ indicates a statistically significant difference from normal group; ҂ indicates a statistically significant difference from CYC group; ¥ indicates a statistically significant difference from CYC + BCP (100 mg/kg) utilizing one-way ANOVA then Tukey’s post hoc analysis (*p* < 0.05).

CYC administration caused numerous ECG abnormalities, including an increase in P-R, R-R, Q-T intervals as well as a decrease in the R wave voltage and heart rate revealing abnormal cardiac function and activity as shown in Table 2 and Figure 1a. On the other hand, animals treated with BCP showed significant attenuation in CYC induced variations in ECG traces (Figure 1a) as revealed by the lowered P-R, R-R, Q-T intervals as well as the increased R wave voltage and heart rate when linked to CYC animals.

### 3.3. β-Caryophyllene Attenuated CYC Induced Fluctuations in Histology of the Cardiac Section

Cardiac sections of the normal and BCP animals displayed normal cardiomyocytes with regular architecture. On the other hand, CYC animals revealed several degenerative changes, including cytoplasmic vacuoles (yellow arrows), necrosis (green arrows) accompanied by scattered mononuclear inflammatory cells (head arrows), myocardial tissue separation, and blood vessel congestion as presented in Figure 1d. BCP administration showed curtailed myofibrils defragmentation, declined myocardium intracellular spaces, and less blood vessel congestion, as shown in Table 3 and Figure 1b,c. Masson’s trichrome sections showed no fibrosis in the control and BCP groups, whereas the CYC group exhibited mild intramuscular fibrosis, as shown in Figure 1b. Furthermore, BCP treated groups showed the restoration of intramuscular fibrosis.

### 3.4. β-Caryophyllene Attenuated CYC Induced Escalation in Cardiac Indices

**Figure 2:**
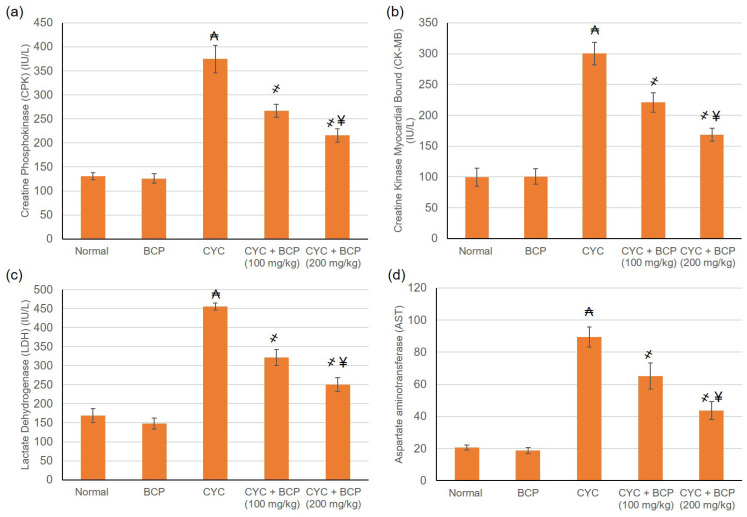
Effects of treatment with β-caryophyllene (BCP) for 14 days on cyclophosphamide (CYC) induced escalation in cardiac indices including (**a**) CPK, (**b**) CK-MB, (**c**) LDH and (**d**) AST in rats. All values are expressed as mean ± SD. ₳ indicates a statistically significant difference from normal group; ҂ indicates a statistically significant difference from CYC group; ¥ indicates a statistically significant difference from CYC + BCP (100 mg/kg) utilizing one-way ANOVA then Tukey’s post hoc analysis (*p* < 0.05).

CYC induced cardiac injury was demonstrated by the intensification in cardiac indices. Cardiotoxicity indices, including CPK, CK-MB, LDH, and AST activities, were substantially intensified in the CYC experienced animals, when related to the normal animals. On the other hand, the daily administration of BCP 100 and 200 mg/kg to CYC challenged rats resulted in a clear reversal of CYC-induced increase in CPK, CK-MB, LDH, and AST activities, as shown in Figure 2.

### 3.5. β-Caryophyllene Attenuated CYC Induced Escalation in Lipid Peroxidation and Oxidative Stress

CYC induced cardiac lipid peroxidation and oxidative stress as evidenced by MDA and H_2_O_2_ elevation as well as deteriorated antioxidant enzymes activities. Treatment with BCP resulted in lipid peroxidation mitigation as shown by the depressed MDA and antioxidant capacity intensification, as revealed by augmented GPx, SOD, CAT, and GRx activities and lowered H_2_O_2_ (Table 4). There was no significant difference between the two doses of BCP (100 and 200 mg/kg) in lowering the lipid peroxidation effect.

### 3.6. β-Caryophyllene Attenuated CYC Induced Reduction in Nrf2 Pathway

**Figure 3:**
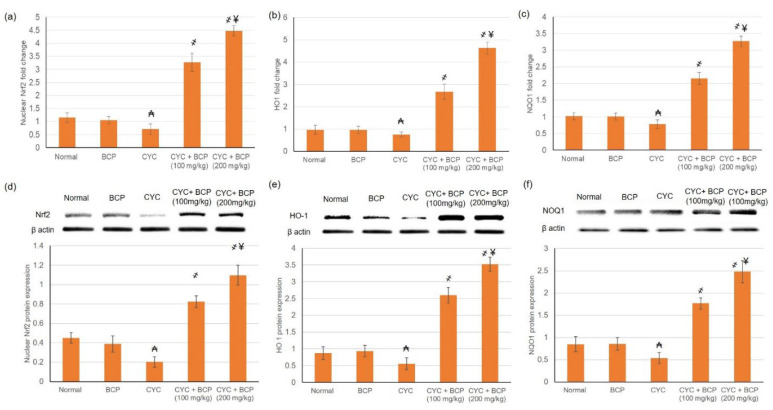
Effects of treatment with β-caryophyllene (BCP) for 14 days on cyclophosphamide (CYC) induced escalation in gene expression of (**a**) Nrf2, (**b**) HO1 and (**c**) NQO1 and protein expression of (**d**) Nrf2, (**e**) HO1 and (**f**) NQO1 in rats. All values are expressed as mean ± SD. ₳ indicates a statistically significant difference from normal group; ҂ indicates a statistically significant difference from CYC group; ¥ indicates a statistically significant difference from CYC + BCP (100 mg/kg) utilizing one-way ANOVA then Tukey’s post hoc analysis (*p* < 0.05).

Real time PCR and Western blot were implemented to study the effect of BCP on gene and protein expression of Nrf2/HO1/NQO1 pathway. Treatment with BCP resulted in an escalation in nuclear Nrf2 mRNA and protein expression, indicating intensified nuclear translocation of Nrf2, with subsequent HO1 and NQO1 mRNA and protein amplification (Figure 3).

### 3.7. β-Caryophyllene Attenuated CYC Induced Escalation in the TLR4 and NFKB

**Figure 4:**
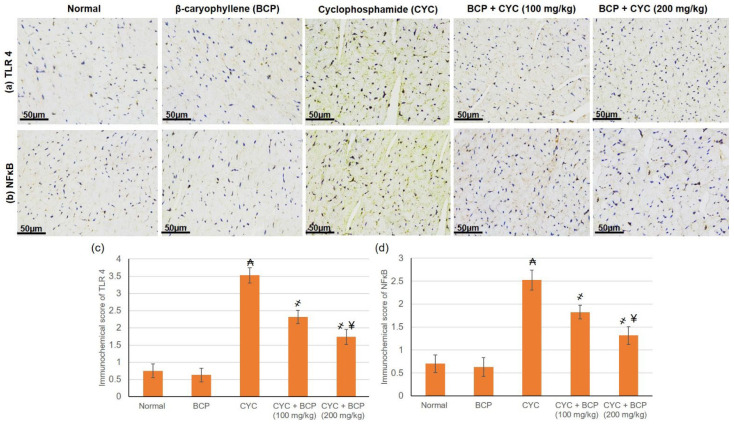
Effects of treatment with β-caryophyllene (BCP) for 14 days on cyclophosphamide (CYC) induced expression of (**a**) toll-like receptors 4 (TLR 4) and (**b**) NFκB by immunohistochemical staining in cardiac tissues, (**c**,**d**) showing semiquantitative analysis of TLR 4 and NFκB immunohistochemical staining. All values are expressed as mean ± SD. ₳ indicates a statistically significant difference from normal group; ҂ indicates a statistically significant difference from CYC group; ¥ indicates a statistically significant difference from CYC + BCP (100 mg/kg) utilizing one-way ANOVA then Tukey’s post hoc analysis (*p* < 0.05).

As shown in Figure 4 CYC injection resulted in a significant elevation in the expression of both TLR 4 and NFκB p65 subunit, specified by the intense brown immunostaining, compared to the control group. In contrast, BCP treatment caused a significant reduction in the expression of TLR 4 and NFκB p65 compared to CYC group.

### 3.8. β-Caryophyllene Attenuated CYC Induced Escalation in the Inflammatory Mediators

**Figure 5:**
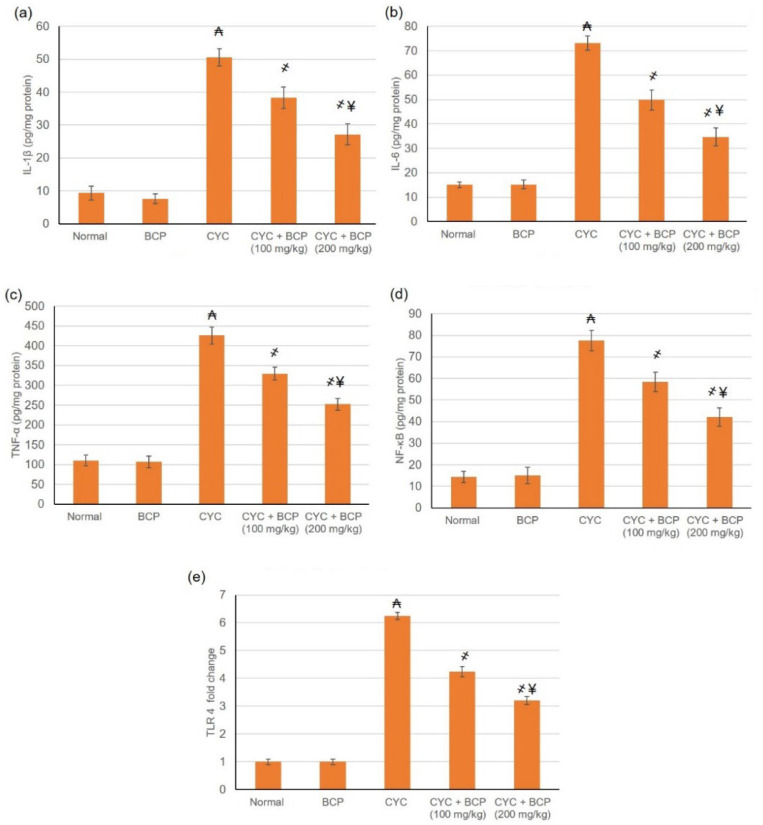
Effects of treatment with β-caryophyllene (BCP) for 14 days on cyclophosphamide (CYC) induced variations on the inflammatory mediators including (**a**) IL-1β (**b**) IL-6 (**c**) TNF-α, (**d**) NFκB and (**e**) TLR 4 mRNA in rat. All values are expressed as mean ± SD. ₳ indicates a statistically significant difference from normal group; ҂ indicates a statistically significant difference from CYC group; ¥ indicates a statistically significant difference from CYC + BCP (100 mg/kg) utilizing one-way ANOVA then Tukey’s post hoc analysis (*p* < 0.05).

TLR 4 mRNA was assessed by real time PCR, whereas NFκB and the inflammatory mediators, including TNF-α, IL-1β, and IL-6, were measured by ELISA. These measures were performed to investigate the anti-inflammatory effect of BCP on CYC induced inflammation within the myocardium via the TLR4/NFκB signaling pathway. CYC induced the overexpression of toll-like receptors 4 (TLR 4) mRNA and NFκB level. Additionally, CYC induced detrimental cardiac inflammation, as evidenced by the increased levels of TNF-α, IL-1β, and IL-6, as shown in Figure 5a–d. On the other hand, management with BCP resulted in a lowered level of TLR 4mRNA expression and NFκB, TNF-α, IL-1β, and IL-6 levels, indicating restricted inflammation. BCP’s anti-inflammatory action may be mediated though inhibiting the TLR4/NFκB signaling pathway.

### 3.9. β-Caryophyllene Attenuated CYC Induced Escalation in Apoptotic Markers

**Figure 6:**
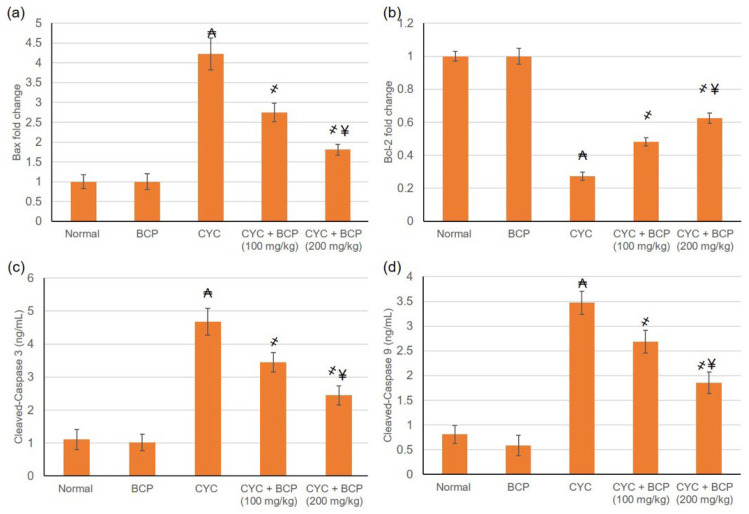
Effects of treatment with β-caryophyllene (BCP) for 14 days on cyclophosphamide (CYC) induced variations on the apoptotic markers including (**a**) Bax, (**b**) Bcl2, (**c**) caspase-3 and (**d**) caspase-9 in rats. All values are expressed as mean ± SD. ₳ indicates a statistically significant difference from normal group; ҂ indicates a statistically significant difference from CYC group; ¥ indicates a statistically significant difference from CYC + BCP (100 mg/kg) utilizing one-way ANOVA then Tukey’s post hoc analysis (*p* < 0.05).

Rats administered CYC only exhibited caspase-3, caspase-9, and Bax proliferation, as well as Bcl2 reduction, signifying apoptosis amplification arising within the myocardium tissue, in comparison with the normal control, as shown in Figure 5. These deviations were significantly alleviated by BCP administration through depressing caspase-3, caspase-9, and Bax and increasing Bcl2 levels with respect to the CYC group (Figure 6), demonstrating the anti-apoptotic efficacy of BCP.

## 4. Discussion

Cardiotoxicity is a major limitation for CYC clinical application as it occurs even within the therapeutic range, resulting in high mortality rate as well as reduced clinical outcome [34]. Additionally, CYC high dose may result in an acute cardiotoxicity in humans, within only ten days, which presents as a mixture of symptoms and signs of myopericarditis leading to fatal complications [35].

In the current study, CYC animals displayed diminished body weight, increased heart to body ratio, numerous ECG irregularities, decreased heart rate, and several degenerative histological changes. Similarly, Bhatt et al. [36] proved that CYC treatment caused an increase in heart weight and decrease in body weight. Body weight reduction may be attributed to the anorexia and food intake reduction [37], whereas increased relative heart weight was related to cardiomyocyte fiber hypertrophy, edema and heart tissues extravasation [35,37]. In addition, the increase in heart weight may be due to extensive necrosis of cardiac muscles and invasion of inflammatory cells to the injured area [36]. Regarding ECG changes, CYC demonstrated prolongation in RR segment, QT segment, PR interval, and QRS interval as well as significant decrease in heart rate [36]. Levine et al. [38] demonstrated that the release of higher quantities of neurotransmitter acetylcholine during myocardial damage causes bradycardia. CYC also induced changes in parasympathetic tone and conduction system via causing an AV block and PR interval prolongation. CYC also cause hyponatremia and hypokalemia, resulting in QT prolongation [39]. In the existing experiment, CYC animals exhibited cardiac indices intensification, which are released into the blood stream because of endothelium injury. Thus, quantitative assessment of these enzymes is related to myocardial injury extend [36]. Similarly, cardiac enzymes were increased in the CYC administered animals in several previous studies [32,35,36]. The underlying cellular mechanisms of CYC meditated cardiac injury include free oxygen radical escalation, inflammation, and apoptosis events [32].

Firstly, with respect to the free oxygen radical escalation, CYC metabolism produces OH free radicals among others, which causes the loss of myocardial membrane integrity and thus its function [36]. Indeed, CYC induced cardiac oxidative stress statues in our study, as evidenced by MDA and H_2_O_2_ elevation as well as deteriorated antioxidant activities. This is in accordance with numerous previous reports that demonstrated a fall in the level of antioxidant enzymes and amplified ROS and lipid peroxidation [34,40]. Nrf2 instigation enhanced antioxidant defenses and diminished oxidative injury, inflammation, and cell death in different organs, e.g., the liver [41], kidney [42], and heart [25], suggesting the Nrf2/HO1/NQO1 pathway as having therapeutic potential for antioxidant defenses intensification. In the existing study, CYC administered animals exhibited depressed Nrf2/HO1/NQO1. In accordance, Hassanein et al. [25] showed that Nrf2 mRNA and protein were decreased significantly in the heart of rats administered with CYC. Nrf2 not only mediates an effect in the cardiac tissue only, but also in other organs. For instance, the Nrf2 pathway ameliorated bladder dysfunction in cyclophosphamide-induced cystitis through oxidative stress suppression [43].

Secondly, regarding the inflammation and apoptosis responses. One of the proposed pathways through which the inflammatory response occurs with CYC is via TLR4/NFκB. CYC induced the overexpression of TLR4 and NFκB accompanied by the overproduction of inflammatory cytokines [28]. Inflammatory cytokine augmentation is one of the reported manifestations of CYC cardiotoxicity [34], causing direct cardiomyocyte damage comprising of further hypertrophy, inflammatory cells infiltration, and widespread necrosis [32]. TNF-α is released during cardiac injury to triggers cardiac dysfunction [44]. Activated macrophages produce IL-1β, which is involved in cell proliferation, differentiation, and apoptosis [45]. The present study revealed that CYC-induced detrimental cardiac inflammation, as evidenced by the increased mRNA expression of TLR4 with subsequent amplified levels of NFκB, TNF-α, IL-1β, and IL-6. A broad spectrum of studies have revealed that inflammatory response of CYC [34,41,42,44]. Furthermore, in the current study, rats administered CYC exhibited caspase 3, 9, and Bax proliferation and Bcl2 reduction, indicating apoptosis amplification arising within the myocardium tissue. Till the moment, there is no approved adjuvant treatment available clinically to mitigate CYC associated cardiac injury. Thus, several natural bioactive compounds have been examined for their potential cardioprotective action against CYC-induced cardiotoxicity. 

β-Caryophyllene (BCP), a natural sesquiterpene, has been utilized as a flavoring agent and perfume component since the 1830s [46]. In the current study, treatment with BCP augmented body weight and decreased heart weight, heart to body weight ratio, reduced heart rate, and amended CYC induced variations in ECG traces and histological sections. Additionally, treatment with BCP at 100 and 200 mg/kg ameliorates CYC induced augmentation in the cardiac enzymes. These outcomes suggested that BCP may preserve the structural integrity of the cardiac cells membranes and subsequently prevent the release of the cardiac enzymes. These results agree with the reported cardiac protective effect of BCP in other cardiac models, including doxorubicin-induced acute cardiac injury [18], isoproterenol induced myocardial infraction [15,16], and diabetic cardiomyopathy [47].

In the present study, treatment with BCP resulted in lipid peroxidation mitigation as well as dose-dependent enriched antioxidant capacity, as revealed by lowered MDA and H_2_O_2_ and enhanced GPx, SOD, CAT, and GRx activities. These results agree with the previously reported antioxidant capacity of BCP in myocardium [16,18], hepatic [48], and renal [49] tissues. Furthermore, treatment with BCP was related with a significant escalation in nuclear translocation of Nrf2, with subsequent HO1 and NQO1 amplification, indicating an intensified Nrf2/HO1/NQO1 signaling pathway. Previously, Li et al. [50] reported that BCP enhanced Nrf2 activation in high glucose (HG)-induced glomerular mesangial cells (MCs). Additionally, via activating the Nrf2/HO1 signaling pathway, BCP mitigated focal cerebral ischemia-reperfusion damage [20], MPTP induced Parkinson’s disease [51], aflatoxin B1 induced liver toxicity [48], and sulfasalazine-induced nephrotoxicity [49].

In the current study, management with BCP was associated with lowered levels of TLR4, NFκB, TNF-α, IL-1β, and IL-6, indicating limited inflammation because of the anti-inflammatory effect of BCP. A wide range of studies documented the anti-inflammatory activity of BCP in different models, e.g., in arthritic [11], skin wound excision [9], sulfasalazine induced nephrotoxicity [49], Mycoplasmal pneumonia [52], and others. BCP depressed caspase-3, caspase-9, and Bax and increased Bcl2 levels, indicating lessened apoptosis state with the myocardium tissue. This result agrees with the work of Al-Taee et al. [18], who reported BCP anti-apoptotic activity. In addition, BCP inhibited Fas-receptor and caspase-mediated apoptosis signaling pathways and endothelial dysfunction in experimental myocardial infarction [16].

This study might experience some limitations. The results only associate the mitigating effect of BCP on the CYC-induced cardiac damage to the studied pathways. Further studies, including mechanistic evaluation using silencing, knockout, overexpression, or/and in vitro experiments is needed to firmly establish the ameliorative effect of BCP on these pathways.

## 5. Conclusions

In conclusion, the results from the present investigation indicate that BCP at 100 and 200 mg/kg shielded the cardiac tissues against CYC induced cardiotoxicity by intensifying antioxidant enzyme activities, and mitigating inflammation, lipid peroxidation, and apoptosis. BCP administration was associated with an intensified Nrf2/HO1/NQO1 pathway, which subsequently enhanced Nrf2-dependent anti-oxidative capacity. Furthermore, management with BCP was related with TLR4/NFκB pathway inhibition, which subsequently diminished the inflammatory response and subdued apoptosis.

## Figures and Tables

**Table 1:** Effects of treatment with β-caryophyllene (BCP) for 14 days on cyclophosphamide (CYC) induced fluctuations in body and heart weights and heart to body weights ratio in rat.

Treatment	Initial Body Weight (gm)	Final Body Weight (gm)	Body Weight Gain (gm)	Heart Weight (gm)	Heart Weight/Final Body Weight (10^3^)
Normal	200.95 ± 15.46	230.43 ± 19.54	29.48 ± 7.16	0.74 ± 0.04	3.21 ± 0.45
BCP	190.63 ± 13.86	225.76 ± 20.45	35.13 ± 6.34	0.73 ± 0.021	3.23 ± 0.35
CYC	212.56 ± 17.67	145.34 ± 10.33 ₳	−67.22 ± 15.85 ₳	0.85 ± 0.06 ₳	5.84 ± 0.95 ₳
CYC + BCP (100 mg/kg)	205.57 ± 16.43	168.23 ± 4.63 ҂	−37.34 ± 8.65 ҂	0.8 ± 0.086 ҂	4.75 ± 0.54 ҂
CYC + BCP (200 mg/kg)	198.34 ± 13.48	174.96 ± 7.23 ҂	−23.38 ± 7.53 ҂¥	0.78 ± 0.067 ҂¥	4.45 ± 0.18 ҂¥

All values are expressed as mean ± SD. ₳ indicates a statistically significant difference from normal group; ҂ indicates a statistically significant difference from CYC group; ¥ indicates a statistically significant difference from CYC + BCP (100 mg/kg) utilizing one-way ANOVA then Tukey’s post hoc analysis (*p* < 0.05).

**Table 2:** Effects of treatment with β-caryophyllene (BCP) for 14 days on cyclophosphamide (CYC) induced fluctuations in ECG and heart rate.

Treatment	P-R Interval (ms.)	R-R Interval (ms.)	Q-T Interval (ms.)	R Wave Amplitude (mV)	Heart Rate (HR) (Beat/min)
Normal	34.53 ± 1.48	148.83 ± 1.36	73.83 ± 1.58	0.27 ± 0.01	435 ± 15.23
BCP	36.76 ± 2.02	146.94 ± 2.02	71.56 ± 2.06	0.25 ± 0.01	421 ± 13.86
CYC	57.98 ± 4.3 ₳	231.76 ± 6.43 ₳	95.21 ± 6.34 ₳	0.15 ± 0.02 ₳	314 ± 27.45 ₳
CYC + BCP (100 mg/kg)	48.16 ± 1.82 ҂	210.16 ± 5.79 ҂	86.29 ± 2.21 ҂	0.21 ± 0.01 ҂	353 ± 12.36 ҂
CYC + BCP (200 mg/kg)	42.11 ± 2.14 ҂¥	184.41 ± 3.43 ҂¥	80.01 ± 2.94 ҂¥	0.23 ± 0.01 ҂¥	374 ± 16.94 ҂¥

All values are expressed as mean ± SD. ₳ indicates a statistically significant difference from normal group; ҂ indicates a statistically significant difference from CYC group; ¥ indicates a statistically significant difference from CYC + BCP (100 mg/kg) utilizing one-way ANOVA then Tukey’s post hoc analysis (*p* < 0.05).

**Table 3:** Effects of treatment with β-caryophyllene (BCP) for 14 days on cyclophosphamide (CYC) induced fluctuations in histology of the cardiac section.

Treatment	Degenerative Changes	Intercellular Spaces	Blood Vessels Congestion
Normal	-	-	-
BCP	-	-	-
CYC	+++	+++	+++
CYC + BCP (100 mg/kg)	++	+	++
CYC + BCP (200 mg/kg)	+	+	+

**Table 4:** Effects of treatment with β-caryophyllene (BCP) for 14 days on cyclophosphamide (CYC) induced augmentation in oxidative stress and lipid peroxidation.

Treatment	MDA (nmole/g Protein)	Glutathione Peroxidase (GPx) (U/g Protein)	Superoxide Dismutase (SOD) (U/g Protein)	Catalase (CAT) (U/g Protein)	Glutathione Reductase (GRx) (nmole/mg Protein)	H_2_O_2_ (nM/g Protein)
Normal	70.66 ± 5.07	300.45 ± 11.51	620.70 ± 32.61	927.33 ± 26.65	89.82 ± 7.81	17.91 ± 1.28
BCP	67.98 ± 8.1	292.05 ± 15.44	601.43 ± 30.24	957.43 ± 25.55	89.98 ± 6.91	18.12 ± 1.51
CYC	172.31 ± 11.45 ₳	68.71 ± 10.45 ₳	174.48 ± 24.75 ₳	250.95 ± 30.24 ₳	24.98 ± 3.65 ₳	38.52 ± 2.13 ₳
CYC + BCP (100 mg/kg)	126.88 ± 12.01 ҂	132.72 ± 14.60 ҂	373.54 ± 20.99 ҂	473.04 ± 34.21 ҂	34.71 ± 4.03 ҂	29.18 ± 1.53 ҂
CYC + BCP (200 mg/kg)	109.52 ± 8.39 ҂	216.14 ± 13.82 ҂ ¥	495.85 ± 33.09 ҂ ¥	590.68 ± 39.84 ҂ ¥	48.87 ± 3.40 ҂ ¥	22.68 ± 1.85 ҂ ¥

All values are expressed as mean ± SD. ₳ indicates a statistically significant difference from normal group; ҂ indicates a statistically significant difference from CYC group; ¥ indicates a statistically significant difference from CYC + BCP (100 mg/kg) utilizing one-way ANOVA then Tukey’s post hoc analysis (*p* < 0.05).

## Data Availability

Not applicable.
